# Association between killer cell immunoglobulin-like receptor (KIR) polymorphisms and systemic lupus erythematosus (SLE) in populations: A PRISMA-compliant meta-analysis

**DOI:** 10.1097/MD.0000000000006166

**Published:** 2017-03-10

**Authors:** Hui-ling Liang, Shu-juan Ma, Hong-zhuan Tan

**Affiliations:** Department of Epidemiology and Statistics, Xiangya School of Public Health, Central South University, Changsha, Hunan, China.

**Keywords:** killer cell immunoglobulin-like receptors, meta-analysis, polymorphism, systemic lupus erythematosus

## Abstract

**Background:**

Recently, a growing number of studies show that the killer cell immunoglobulin-like receptor (KIR) gene polymorphisms may play a role in the systemic lupus erythematosus (SLE) susceptibility. Nonetheless, the results were inconsistent. Thus, a meta-analysis was carried out by integrating multiple research to clarify the association between KIR polymorphisms and SLE susceptibility.

**Methods:**

The Web of Science, Embase (Ovid), PubMed, Elsevier Science Direct, the Chinese Biomedical Database and CNKI, Wanfang databases (last search was updated on May 15, 2016) were systematically searched to select studies on addressing the association between the KIR polymorphisms and susceptibility to SLE in populations. The odds ratio (OR) with 95% confidence interval (95% CI) was calculated.

**Results:**

A total of 10 published case-control studies involving 1450 SLE patients and 1758 controls were available for this meta-analysis. Results suggested that KIR2DL1 might be a risk factor for SLE (OR _2DL1_ =1.047, 95% CI=1.011–1.083) in all subjects. The KIR2DL3, KIR2DL5 were identified as protective factors for SLE in Asian populations (OR_2DL3_= 0.215, 95% CI = 0.077–0.598; OR_2DL5_ = 0.588, 95% CI = 0.393–0.881), but not in Caucasians.

**Conclusions:**

The meta-analysis results suggested that 2DL1 might be a potential risk factor and 2DL3, 2DL5 might be protective factors for SLE in Asians but not in Caucasians.

## 1 Introduction

Systemic lupus erythematosus (SLE) is a systemic autoimmune disease with multiorgan inflammation. It is characterized by production of pathogenic autoantibodies and deposition of immune complexes in multiple organs, reflecting a global loss of self-tolerance,^[1]^ which then result in a wide array of clinical manifestations in skin, blood, kidney, joints, heart and central nervous system, and so on.^[2]^ It usually affects women more often than men in a ratio of 8:1 approximately. The peak age of SLE diagnosis ranges from 15 to 44 years old. Compared with other groups, women of child-bearing age are susceptible to the highest incidence of SLE.^[3]^ The etiology of SLE still remains unclear, but it is generally accepted that the onset of SLE is a multifactorial combined result, involving environmental, genetic, age, and hormonal factors.^[4]^ Aberrant innate immune responses play a significant role in the pathogenesis of SLE, contributing both to tissue injury via release of inflammatory cytokines as well as to aberrant activation of autoreactive T and B cells. Moreover, many studies have identified more than 30 robust genetic associations with SLE including killer cell immunoglobulin-like receptors (KIRs), *IRF5*, *STAT4*, *PTPN22*, *TNFAIP3*, *BLK*, *BANK1*, *TNFSF4,* and *ITGAM*.^[5]^

The KIR receptors are members of the immunoglobulin family present on cell surface, expressed in NK cells21 and some T lymphocytes.^[6]^ The KIR genes are clustered on the KIR locus located in the leukocyte receptor complex spanning a region of about 150 to 200 kb on chromosome 19q13.4.^[7]^ In humans, 17 highly homologous genes have been identified to date: 6 activating (KIR2DS1-5, and KIR3DS1), 9 inhibitory (KIR2DL1-4, KIR2DL5A, KIR2DL5B, and KIR3DL1-3), and 2 pseudogenes (KIR2DP1 and KIR3DP1).^[8]^ Recent studies have shown strong associations between KIR and human leukocyte antigen genes with susceptibility to autoimmune diseases such as rheumatoid arthritis,^[9,10]^ multiple sclerosis,^[11]^ SLE,^[12,13]^ type I diabetes (T1DM),^[14,15]^ and type II diabetes (T2DM),^[16,17]^ etc.

The association between KIR gene polymorphisms and SLE risk has been investigated by many case-control studies, but findings are not always consistent. Many of which were due to false-positive/negative studies, true variability in different populations, small sample sizes, low statistical power, or clinical heterogeneity are potential factors that could result in the discrepancies in these studies.^[18]^ To overcome the limitations on this issue, meta-analysis is an efficient method, which can integrate many previous small research studies and increase statistical power by pooling the results of single study.^[19]^ We performed this meta-analysis for the purpose of investigating the exact relationships between KIR polymorphisms and susceptibility to SLE.

## 2 Materials and methods

### 2.1 Ethnic statement

The meta-analysis was based on previous published studies, thus no ethical approval and patient consent are required.

### 2.2 Literature search strategy

This meta-analysis was performed according to the Preferred Reporting Items for Systematic Reviews and Meta-Analyses (PRISMA) guidelines.^[20]^ The following electronic databases were searched: the Web of Science, Embase (Ovid), PubMed, Elsevier Science Direct, the Chinese Biomedical Database (CBM) and CNKI, and Wanfang databases (last search was updated on May 15, 2016), using the following search terms:“systemic lupus erythematosus” or “SLE,” “The killer cell immunoglobulin-like receptors” or “KIR” or “KIRs,” “polymorphism” or “gene” or “genes” or “genotype” or “genotypes” or “genotyping” or “single nucleotide” or“allele.” All searched studies were retrieved, and their references were also scanned to identify additional relevant studies.

### 2.3 Inclusion and exclusion criteria

Studies fulfilling the following inclusion criteria were qualified for this meta-analysis: case–control study; articles investigating the association between KIRs and SLE risk; sufficient published data available for estimating the odds ratio (OR) with 95% confidence interval (CI); and at least 2 comparison groups (SLE patient group vs control group) involved in a single study. The controls’ ethnic background and geographic area were comparable with the cases’. The publication was in English or Chinese. Exclusion criteria were as follows: research that did not meet the inclusion criteria; the study reported duplicated or useless data.

### 2.4 Quality assessment

Newcastle–Ottawa scale (NOS) criteria was used to assess the methodological quality of all included studies.^[21]^ This scale contains 9 items in total (1 point for each) in 3 parts: subject selection: 0 to 4; subject comparability: 0 to 2; exposure: 0 to 3. Study with NOS scores ≥7 was regarded as good quality (range, 0–9).

### 2.5 Data extraction

Two investigators separately extracted the data with the standard protocol, any disagreement was addressed by discussion and resolved by consultation with the third investigator. The following items were extracted: first author, publication year, country of origin, ethnicity, genotyping method, KIR gene polymorphisms. The information is shown in Table 1. The NOS was used to assess the quality of the included studies.

**Table 1:**
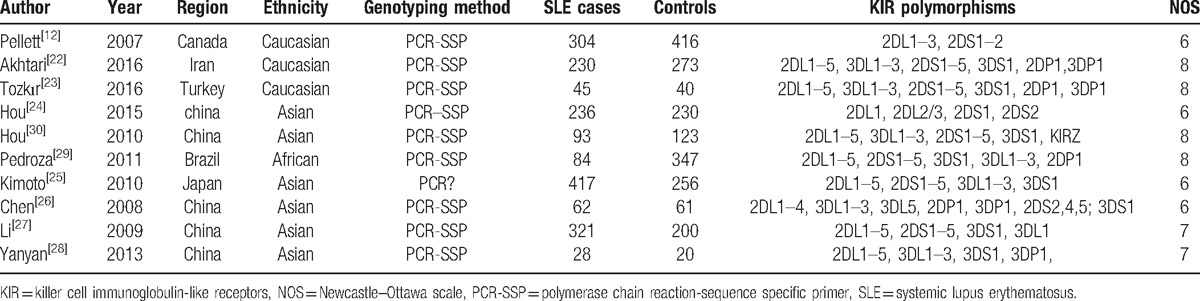
Characteristics of individual studies included in meta-analysis.

### 2.6 Statistical analysis

The strength of associations between KIR gene polymorphisms and SLE risk was assessed by OR with the corresponding 95% confidence intervals (CI), *P* value <0.05 was deemed to be statistically significant. Heterogeneity was measured using the Q statistic, and *P* value < 0.1 was considered statistically significant. The extent of heterogeneity was further quantified using the *I*^2^ statistic, *I*^2^ values of 25%, 50% and 75% were defined as low, moderate, and high heterogeneity, respectively. When the *P* value was >0.10 or *I*^2^ value was <50%, the pooled OR was calculated using the fixed-effect model; otherwise, a random-effect model was used. The *χ*^2^ test was applied to examine whether the genotype distributions in the control group of each study were in Hardy–Weinberg equilibrium. Furthermore, we performed funnel plots and Egger test assessed potential publication bias, when *P* value <0.05 was considered statistically significant. Subgroup analyses were conducted by ethnic group. Sensitivity analysis was performed by excluding individual study to assess the stability of the results. All data analysis was performed with STATA 12.0 software.

## 3 Results

### 3.1 Studies included in the meta-analysis

**Figure 1:**
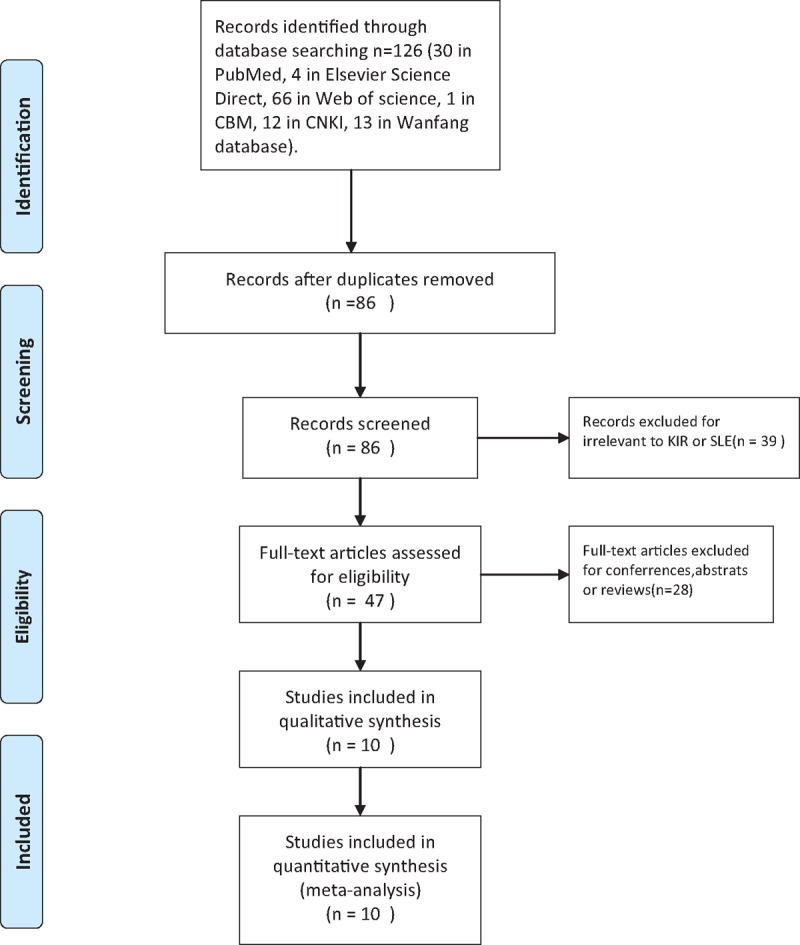
Flow diagram of study selection.

The search strategy retrieved 126 potentially relevant studies (30 in PubMed, 4 in Elsevier Science Direct, 66 in Web of Science, 1 in CBM, 12 in CNKI, 13 in Wanfang database). According to the inclusion criteria, 10 articles were included in this meta-analysis, which contained a total of 1450 SLE cases and 1758 healthy controls. A flow diagram of study selection process is illustrated in Fig. 1. All studies were case-control studies that investigated the association of KIR polymorphisms and susceptibility to SLE. Among the 10 articles, 3^[12,22,23]^ were Caucasians, 6^[24–28]^ were East Asians, 1^[29]^ was South Americans. Owning to the insufficient sample populations available for the South American group, we conducted ethnicity-specific meta-analyses for Caucasian and Asian populations. Characteristics of the included studies are summarized in Table 1. Six^[21,22,24,25,28,29]^ of 10 studies were of high quality (NOS score ≥7) and the other 4 studies were defined as moderate quality (all scored 6), which are shown in Table 1.^[12,22–30]^

### 3.2 Main results

**Figure 2:**
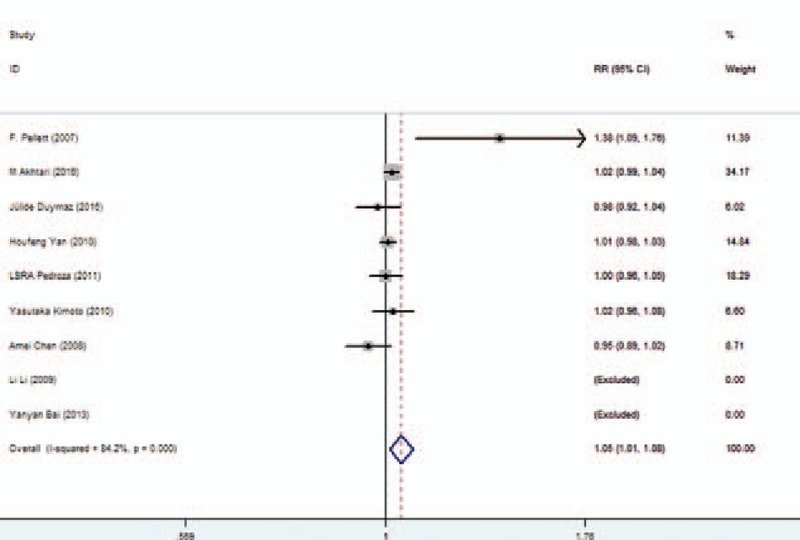
Association between 2DL1 and susceptibility to SLE in all subjects. SLE = systemic lupus erythematosus.

Meta-analysis of KIR polymorphisms and SLE are listed in Table 2. Fifteen KIR polymorphisms were addressed in 10 case-control studies: 2DL1, 2DL2, 2DL3, 2DL4, 2DL5, 3DL1,3DL2, 3DL3, 2DS1, 2DS2, 2DS3, 2DS4, 2DS5, 3DS1, 3DP1. The most common polymorphisms were 2DL3 and 3DL1. Meta-analysis results suggested 1 positive association of 2DL1 with susceptibility to SLE (OR _2DL1_ =1.047, 95% CI=1.011–1.083) (Fig. 2). However, no association had been found between 2DL2–5, 3DL1–3, 2DS1–5, 3DS1, 3DP1 and susceptibility to SLE (Table 2).

**Table 2:**
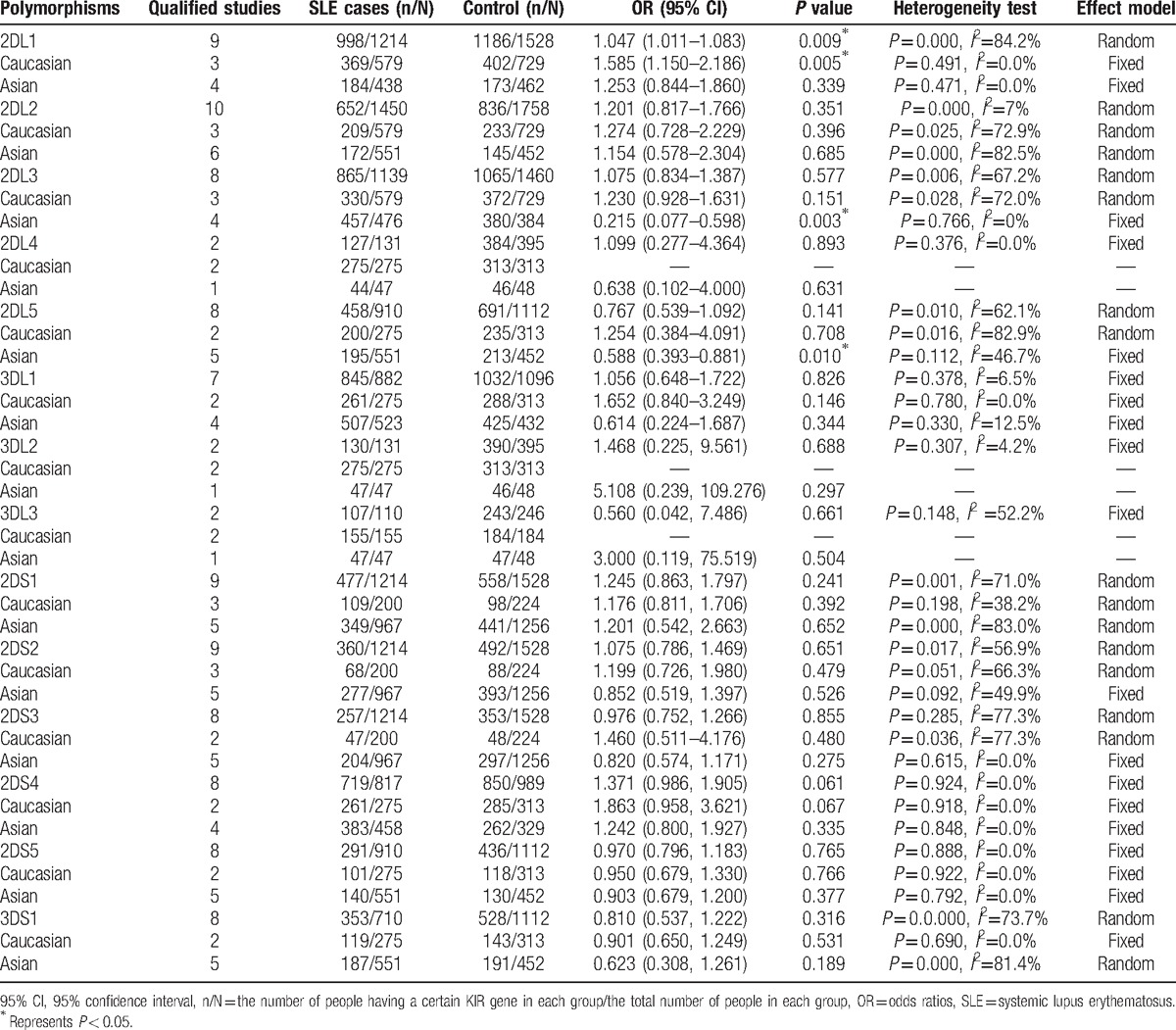
Meta-analysis of the association between KIR polymorphisms and SLE.

### 3.3 Heterogeneity test, subgroup, and sensitivity analysis

In our results, significant heterogeneity (*I*^2^ > 50%) between studies was found in 2DL2, 2DL3, 2DL5, 2DS1–3, 3DS1, but heterogeneity in other KIR polymorphisms was not observed. Thus, the pooled OR was calculated by random effect model and fixed effect model, respectively.

**Figure 3:**
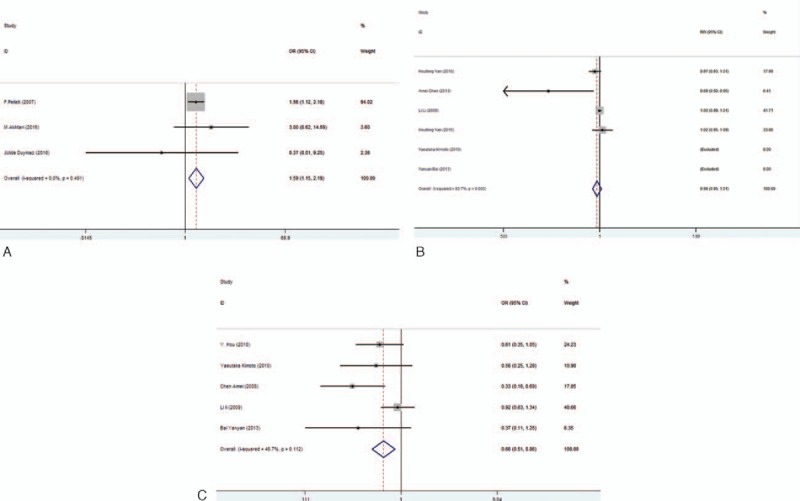
A, Association between 2DL1 and susceptibility to SLE in Caucasians. B, Associations between 2DL3 and susceptibility to SLE in Asians. C, Associations between 2DL5 and susceptibility to SLE in Asians. SLE = systemic lupus erythematosus.

Because of the existence of heterogeneity, subgroup analyses were performed to investigate the potential effect of ethnicity in the Asian and Caucasian populations. Our results showed that there was positive association between 2DL1 (fixed-effect model: OR_2DL1_ = 1.585, 95% CI =1.150–2.186) and susceptibility to SLE in Caucasians. Meanwhile, there were 2 negative associations between 2DL3, 2DL5 (fixed-effect model: OR_2DL3_= 0.215, 95% CI = 0.077–0.598, fixed-effect model: OR_2DL5_ = 0.588, 95% CI = 0.393–0.881), and SLE in Asians (Fig. 3A–C). Nevertheless, 2DL2, 2DL4, 3DL1–3, 2DS1–5 showed no association with susceptibility to SLE both in Caucasians and in Asians (Table 2).

**Figure 4:**
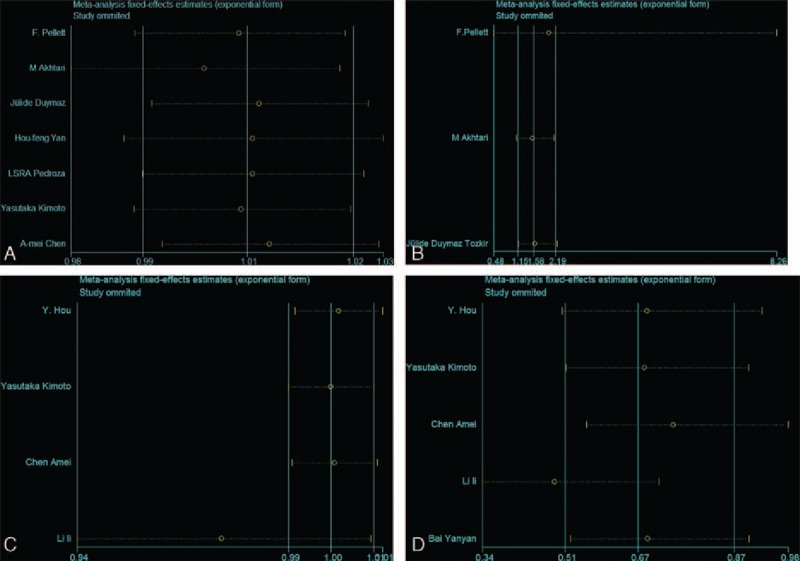
A, Sensitivity analyses between 2DL1 and susceptibility to SLE in all subjects. B, Sensitivity analyses between 2DL1and susceptibility to SLE in Caucasians. C, Sensitivity analyses between 2DL3 and susceptibility to SLE in Asians. D, Sensitivity analyses between 2DL5 and susceptibility to SLE in Asians. SLE = systemic lupus erythematosus.

**Figure 5:**
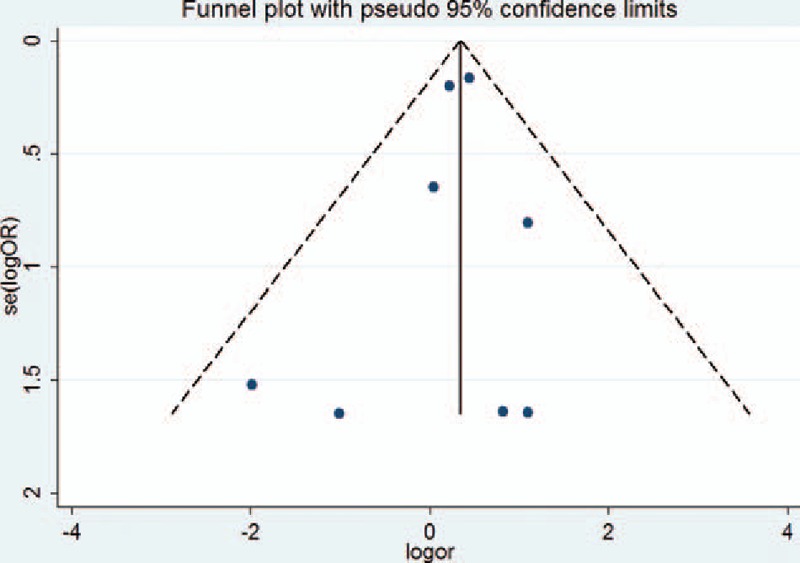
Forest plot of the association between KIR and susceptibility to SLE. KIR = killer cell immunoglobulin-like receptors, SLE = systemic lupus erythematosus.

We further conducted sensitivity analyses by omitting 1 study every time from pooled analysis to examine whether our final results were affected by any single study (Fig. 4A–D). For KIR 2DL2 and SLE, after removing the Chen Amei et al study,^[27]^*P* = 0.325 changed to *P* < 0.0001, so the results need caution. Other corresponding pooled ORs were not substantively altered. Publication bias was evaluated by funnel plot and Egger test on 2DL1. The graphical funnel plots of 6 studies appeared to be symmetrical, no significant publication bias was observed in our meta-analysis. (Egger regression test: *t*=0.42, *P* = 0.744, 95% CI = −0.268 to 0.286) (Fig. 5).

## 4 Discussion

SLE is a multifactorial and highly polymorphic systemic autoimmune disease that predominantly afflicts women in child-bearing age; it is a complex interaction result of genetic, environmental, and hormonal factors, companying a global loss of immune tolerance.^[31]^ In recent years, the genetic basis of SLE has been advanced remarkably through genome-wide association studies in diverse populations.^[32]^ NK cells participate in the immune response and T cell activation. They express inhibitory and activating killer-cell immunoglobulin-like receptor that identifies human leukocyte antigen class I molecules.^[33]^

In 2007, Pellett et al first reported that the frequency of KIR 2DS1 was significantly increased in SLE patients compared with controls.^[23]^ Since then, the association between KIR polymorphism and SLE susceptibility has been replicated in many studies, and some may be inconsistent and population specific. Thus, we performed this current meta-analysis quantitatively so as to evaluate the association between KIR polymorphisms and SLE. Finally, 10 separate case-control studies focusing on the association of KIR to SLE risk were included comprising a total of 1450 SLE cases and 1758 healthy controls. The pooled results showed 1 positive association of 2DL1, so 2DL1 might be a potential risk factor for SLE in multiple ethnic populations. However, we failed to find correlations between other KIR genes with susceptibility to SLE (*P* > 0.05). It is worth noting that in the sensitivity analysis of 2DL2, after omitting the Chen et al study,^[26]^ the result had substantially alteration and the findings should thus be treated with caution.

Obvious heterogeneity between studies was detected, which may be attributed to different characteristics in different studies, quality of each research, different experimental designs and different genotyping methods, different geographic distributions with different genetic backgrounds.^[34]^ Therefore, we performed a subgroup analysis by ethnicity to identify the origin of heterogeneity. In the subgroup analyses, the results showed that 2DL1 might be a risk factor in Caucasians but not in Asians. Furthermore, the results also suggested 2DL3, 2DL5 might be protective factors in Asians but not in Caucasians, indicating that the association between KIR polymorphisms and the risk of SLE may be different in different ethnic populations. Various ethnic and environmental factors may influence susceptibility to disease, which is consistent with previous studies.^[35,36]^ No evidence showed publication bias in this meta-analysis.

Several limitations of our meta-analysis should be noted. First, we were unable to conduct further analysis to explore other risk factors due to the insufficient original data, such as environment–gene/gene–gene interaction, impact of different clinical subtypes or different course of SLE, and so on.^[37]^ Second, we could not perform further subgroup analysis other than ethnicity, and even in the ethnic-specific subgroup analysis, we could only pool the results of Caucasians and Asians, respectively; south Africans were not possible due to limited data, and the division criteria of ethnic groups was crude, which may constrain the general application of our findings. Third, publication bias might occur for only including published articles, some relevant unpublished studies with null results were missed, Although we performed Egger regression test, we still could not remove the possibility of bias. Although our study has above limitations, this is the first meta-analysis focusing on the correlation between KIR gene polymorphisms and susceptibility to SLE. More importantly, we established a rigorous protocol and performed a statistical approach to integrate these inconsistent results through the whole process.^[38]^ Thus, the reliability of the results is guaranteed.

In conclusion, this meta-analysis shows 2DL1 might be a potential risk factor for SLE in Caucasians and 2DL3, 2DL5 might be protective factors for SLE in Asians. Thus, these genes may be applied to clinical early diagnosis of SLE as practical biomarkers.^[39]^ Due to the above limitations, more research studies with larger sample sizes and more detailed information are needed to further validate and confirm our results.

## Acknowledgments

The authors thank all the anonymous reviewers and editors for their suggestions, which will be helpful for the authors to improve their paper.
